# Xenotransplantation of Human Adipose-Derived Stem Cells in Zebrafish Embryos

**DOI:** 10.1371/journal.pone.0123264

**Published:** 2015-04-07

**Authors:** Jin Li, Guofang Zeng, Yawei Qi, Xudong Tang, Jingjing Zhang, Zeyong Wu, Jie Liang, Lei Shi, Hongwei Liu, Peihua Zhang

**Affiliations:** 1 Institute of Plastic Surgery, Affiliated Hospital of Guangdong Medical College, Zhanjiang, Guangdong Province, China; 2 Institute of Biochemistry and Molecular Biology, Guangdong Medical College, Zhanjiang, Guangdong Province, China; 3 Clinical Research Center, Affiliated Hospital of Guangdong Medical College, Zhanjiang, Guangdong Province, China; 4 School of Light Industry and Food Science, South China University of Technology, Guangzhou, Guangdong Province, China; 5 Department of Plastic Surgery, the First Affiliated Hospital of Jinan University, Key Laboratory for Regenerative Medicine, Ministry of Education, Guangzhou, Guangdong Province, China; National University of Singapore, SINGAPORE

## Abstract

Zebrafish is a widely used animal model with well-characterized background in developmental biology. The fate of human adipose-derived stem cells (ADSCs) after their xenotransplantation into the developing embryos of zebrafish is unknown. Therefore, human ADSCs were firstly isolated, and then transduced with lentiviral vector system carrying a green fluorescent protein (GFP) reporter gene, and followed by detection of their cell viability and the expression of cell surface antigens. These GFP-expressing human ADSCs were transplanted into the zebrafish embryos at 3.3–4.3 hour post-fertilization (hpf). Green fluorescent signal, the proliferation and differentiation of human ADSCs in recipient embryos were respectively examined using fluorescent microscopy and immunohistochemical staining. The results indicated that human ADSCs did not change their cell viability and the expression levels of cell surface antigens after GFP transduction. Microscopic examination demonstrated that green fluorescent signals of GFP expressed in the transplanted cells were observed in the embryos and larva fish at post-transplantation. The positive staining of Ki-67 revealed the survival and proliferation of human ADSCs in fish larvae after transplantation. The expression of CD105 was observable in the xenotransplanted ADSCs, but CD31 expression was undetectable. Therefore, our results indicate that human ADSCs xenotransplanted in the zebrafish embryos not only can survive and proliferate at across-species circumstance, but also seem to maintain their undifferentiation status in a short term. This xenograft model of zebrafish embryos may provide a promising and useful technical platform for the investigation of biology and physiology of stem cells *in vivo*.

## Introduction

Human embryonic stem cells (ESCs) and induced pluripotent stem cells (iPSCs) have great developmental potential for treatment in various fields of cell therapy and regenerative medicine [1–3], but ethical problems of ESCs and poor efficient induction of iPSCs both limit their use. But human adipose-derived stem cells (ADSCs) from adults have fewer ethical concerns, and they have strong capacities of differentiation into adipogenic, osteogenic, chondrogenic, neuronal, and myogenic lineages [4–6], and therefore, become an alternative source for other pluripotent cells.

Generation of animal chimerism via cell transplantation is a useful tool for the research of engraftment, expansion, differentiation, and plasticity of allo- or xenogeneic stem cells *in vivo* [7]. Xenograft at fetal stage of the recipient contributes to diminishing the immunological rejection [8–9]. Various chimerisms via xenografts of stem cells have been established with sheep, goats, rats, and pigs in the past years [10–13]. In recent years, zebrafish as one of the most important vertebrate model systems, has become a widely used animal model for study in developmental biology, genetics, neurobiology and regenerative medicine [14–15]. It has reported that human metastatic melanoma cells transplanted into zebrafish blastula-stage embryos did not form tumors nor integrate into host organs, but retained their dedifferentiated phenotype, and furthermore could survive, divide and exhibit motility [16]. Cultured melanocytes and fibroblasts also survived in zebrafish embryos, but malignant melanoma cells are more migratory compared with these normal human cell types [16]. These findings suggest that malignant melanoma cells might respond to environmental cues present in zebrafish embryos that could influence the phenotype and behavior of human cells. Another study indicated that cancer cells grown in mammospheres from breast carcinoma cell lines migrated to the tail of the embryo after transplantation, and formed masses with a significantly higher frequency than parental monolayer populations [17]. Additionally, zebrafish transplantation models have also been widely used for the study of human cancers [18–20]. During the last few years, many zebrafish models have been generated to study cancer heterogeneity, tumor initiation, progression, metastasis, relapse and to screen or test new drug candidates as *in vivo* systems [21–24]. In addition, rapid embryonic development and transparent embryo of zebrafish at their early stages allow researchers to directly observe the morphogenesis of tissues and organs, making them become an ideal recipient model for investigation in stem cell xenografts.

Currently, little is known regarding their fate *in vivo* after xenografts of human stem cells in zebrafish embryos. Therefore, in the present study, we have established a zebrafish model for cell xenotransplantation of human ADSCs expressing green fluorescent protein (GFP) gene to investigate whether these cells would proliferate and differentiate *in vivo*. This xenograft model in zebrafish embryos is a useful technical platform for investigation of the biology and physiology of stem cells *in vivo*.

## Materials and Methods

### Isolation and culture of human adipose-derived stem cells

The isolation and culture of human ADSCs were performed as we described previously [25], and the ADSCs at passages 4–8 were used for subsequent experiments. These studies including the written informed consents were reviewed and approved by the Human Research and Ethical Committee of Affiliated Hospital of Guangdong Medical College. Furthermore, the written informed consents were obtained from the participants who each one donated about 2 g subcutaneous adipose tissue for the isolation of stem cells.

### Lentivirus-mediated transduction of ADSCs

Cell transduction were performed as described previously [26–27], with some modifications. Briefly, ADSCs at passage 4 were seeded in 6-well plates in Dulbecco’s Modified Eagle’s Medium (DMEM) culture medium added with 10% fetal bovine serum (FBS) (Gibco, Carlsbad, CA). Upon reaching 70% cell confluence, the cells were incubated in 1 mL of DMEM medium containing 200 μL lentivirus particles that carry GFP gene at an ideal multiplicity of infection (MOI) of 200 with high efficiency. After 24 h, culture medium was replaced with 2 mL of fresh culture medium, and transduction efficiency was assessed by monitoring GFP expression under fluorescent microscopy. To obtain a cell population expressing GFP, the cells were maintained in DMEM medium added puromycin (1 μg/mL) for screening. GFP-expressing ADSCs were used for the following experiments.

### Cell viability assay

MTT was used to evaluate cell viability 72 h after cell transduction. Briefly, control cells (ADSCs) that were not transfected by lentivirus particles or GFP-expressing ADSCs were seeded into 96-well plates at a density of 1 × 10^4^ cells/well. After culture of 24, 48, and 72 h, 20 μL of MTT (3-[4, 5-dimethylthiazol-2-yl]-2, 5-diphenyltetrazolium bromide) solution (5 mg/mL) was added into each well for another incubation of 4 h, and followed by removing the culture medium and, 150 μL of dimethyl sulfoxide (DMSO) was added to dissolve crystals. The value of optical density at 570 nm was determined with automated spectrophotometric plate reader (PerkinElmer, USA).

### Cell surface antigens of ADSCs

After transduction of 72 h, cell surface marker of ADSCs was determined with flow cytometry as we described previously [25]. Briefly, the control ADSCs and GFP-expressing ADSCs were separately were harvested, and incubated with FITC or APC or PE-conjugated primary monoclonal antibodies against CD44, CD45, CD105, CD29, CD34, CD90, CD13, CD106, CD31, and CD14 (BD Bioscience, CA) for 30 min, and CD surface markers were then assayed on a Becton Dickinson FACSCalibur flow cytometer by using CELLQestPro acquisition software (Becton-Dickinson, CA, USA). Nonspecific IgG isotype control was used to detect background fluorescence.

### Cell transplantation

**Fig 1:**
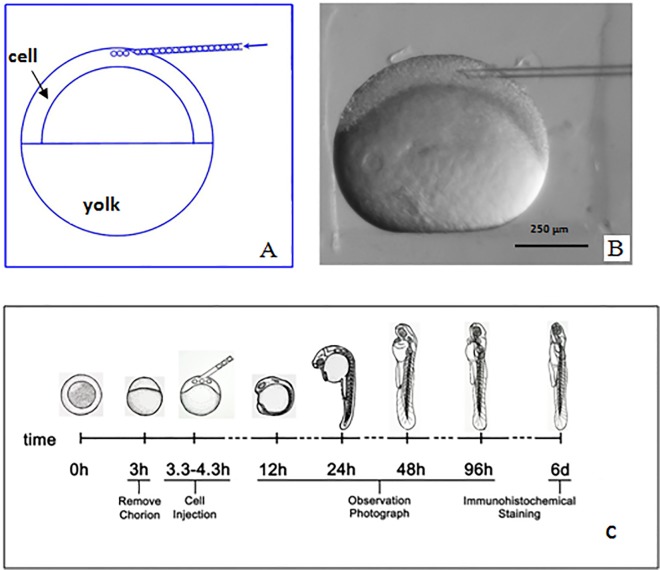
The structure of zebrafish embryo and experimental design. (A) Sketch of zebrafish embryo at selected stage. (B) The image of cells-injected dechorionated zebrafish embryo at 4.3 hpf. (C) We drew schematic representation of experimental design.

Wild-type zebrafish (*Danio rerio*) strain (AB) were raised and maintained according to standard procedures [28]. Each embryo chorion was carefully removed 3 h after fertilization with forceps under stereomicroscope. GFP-expressing human ADSCs were then transplanted into 200 zebrafish embryos at 3.3–4.3 hour post-fertilization (hpf) using glass needle under micromanipulator, as shown in Fig 1. Each embryo was injected with about 10 cells expressed GFP, and the control embryos did not receive cell transplantation. After transplantation, zebrafish embryos were maintained in 6-well plates for their development at 28°C, and observed using fluorescence microscope or laser confocal fluorescence microscope (Leica TCS SP8, Germany) at various time points.

### Immunohistochemical staining

Immunohistochemical staining of zebrafish was performed to investigate the proliferation and differentiation of transplanted ADSCs. The fish were fixed in 2% paraformaldehyde at 4°C overnight at 2 or 6 days post fertilization, and pre-embedded in the 1% agar solution under stereomicroscope. After solidification, the fish were gradually dehydrated with 80%, 90%, and 95% ethanol for 1 h, and 100% ethanol for 30 min, and followed by treatment with xylene for two times, each for 5 min. Subsequently, the fish were embedded in paraffin, and sectioned into 5 μm thick slices. The endogenous peroxidase activity was depleted by adding hydrogen peroxide and blocking with normal serum for 30 min. Then, the sections were separately incubated with rabbit anti-human Ki-67(ZSGB-BIO, Beijing, China), rabbit anti-human CD105 and CD31 (Boster, Wuhan, China) monoclonal antibodies overnight at 4°C, and followed by incubation with biotinylated anti-rabbit IgG secondary antibody for 20 min. Negative control was made without the primary antibody. The sections were then respectively incubated with avidin-biotin-peroxidase complex (Vector Laboratories Ltd., Peterborough, UK) for 1 h, and with 3, 3-diaminobenzidine (Sigma-Aldrich, USA) for 20 min for visualization. The sections were finally counterstained with hematoxylin. The images were captured under the microscope with a digital camera.

### Immunofluorescence staining

To investigate the proliferation and differentiation of transplanted ADSCs in vivo, the immunofluorescence staining of whole-mount zebrafish larvae or the tissue sections of fish were performed, respectively. In brief, the 40 zebrafish embryos of wild-type strain AB at 3.3–4.3 hpf were carefully chorioned, and some of them (20) served the control, and the other aliquots (20) were injected with GFP-expressing human ADSCs at a dose of 10 cells per embryo. The zebrafish larvae were then fixed in 2% PFA and 0.5% Triton X-100 over night at 4°C for the immunofluorescence staining of whole-mount fish after 48 h of cell transplantation. Subsequently, the larvae were washed three times with PBST (PBS, 0.5% Triton X-100, pH7.4), and then blocked with PBST (PBST, 0.2% Triton X-100, 5% NGS, 1% BSA) for 1 h at room temperature with lightly shake. The larvae were then incubated in the blocking solution containing the primary antibody Ki-67 (1:100) over night at 4°C, and followed by washing five times with PBST for 30 min each, and then incubated with the secondary antibody (S10 a-M-cy5, 1: 200) for 3 h at room temperature, and followed by washing five times with PBST for 30 min each. Finally, the zebrafish larvae were observed with laser confocal scanning microscope (Leica TCS SP8, Germany) using a standard parameter for green or red fluorescence. The immunofluorescence of tissue sections was performed to detect the expression of CD105 and CD31 in the transplanted ADSCs. Briefly, the zebrafish were fixed in 4% paraformaldehyde at 4°C overnight, and rehydrated gradually. After embedment in paraffin, the fish were sectioned into 5 μm thick slices, which were incubated with primary antibodies (Anti-CD105, 1:150, anti-CD31, 1: 150) in blocking solution, and then washed several time with PBST, and followed by incubation in secondary antibodies (S10 a-M-cy5, 1: 300) for 2h. After washing three times, the tissue slices were observed with laser confocal scanning microscope, and the images were captured.

## Results

### Morphological features of human ADSCs and transduction

**Fig 2:**
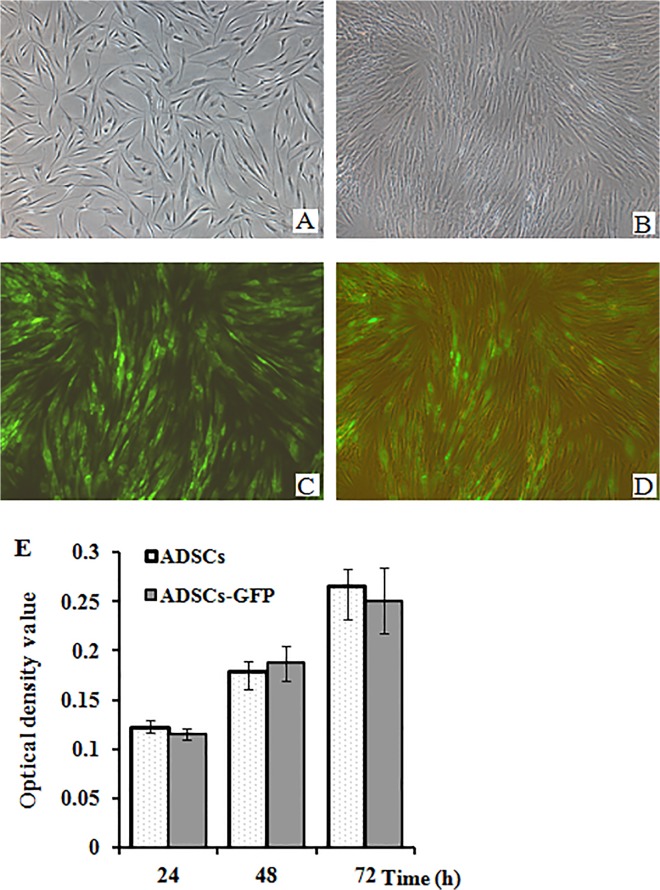
Expression of GFP in human ADSCs. Human ADSCs were isolated from human adipose tissues and transduced with lentivirus vectors carrying GFP reporter gene. (A) spindle-like appearance of primary ADSCs at passage 3. (B) Phase-contrast microscopic image. (C) Fluorescence microscopic image. (D) merged image of (B) and (C). Original magnification of all images: 40 ×. (E) The effect of GFP expression on the proliferation of ADSCs.

Human primary ADSCs were obtained from human adipose tissues, and they demonstrated a fibroblast-like spindle shape (Fig 2A), similar to bone marrow- derived mesenchymal stem cells, and proliferated quickly in the culture medium. Before cell transplantation, ADSCs were transduced with lentivirus vector system carrying GFP reporter gene prior. The results indicated that more than 90% of ADSCs were positive for GFP expression after transduction and screening (Fig 2B–2D). In addition, exogenous GFP expression in ADSCs did not change cell morphology and cell viability (Fig 2E), which was in according with previous reports [29].

### Cell surface antigens detected by flow cytometry

**Fig 3:**
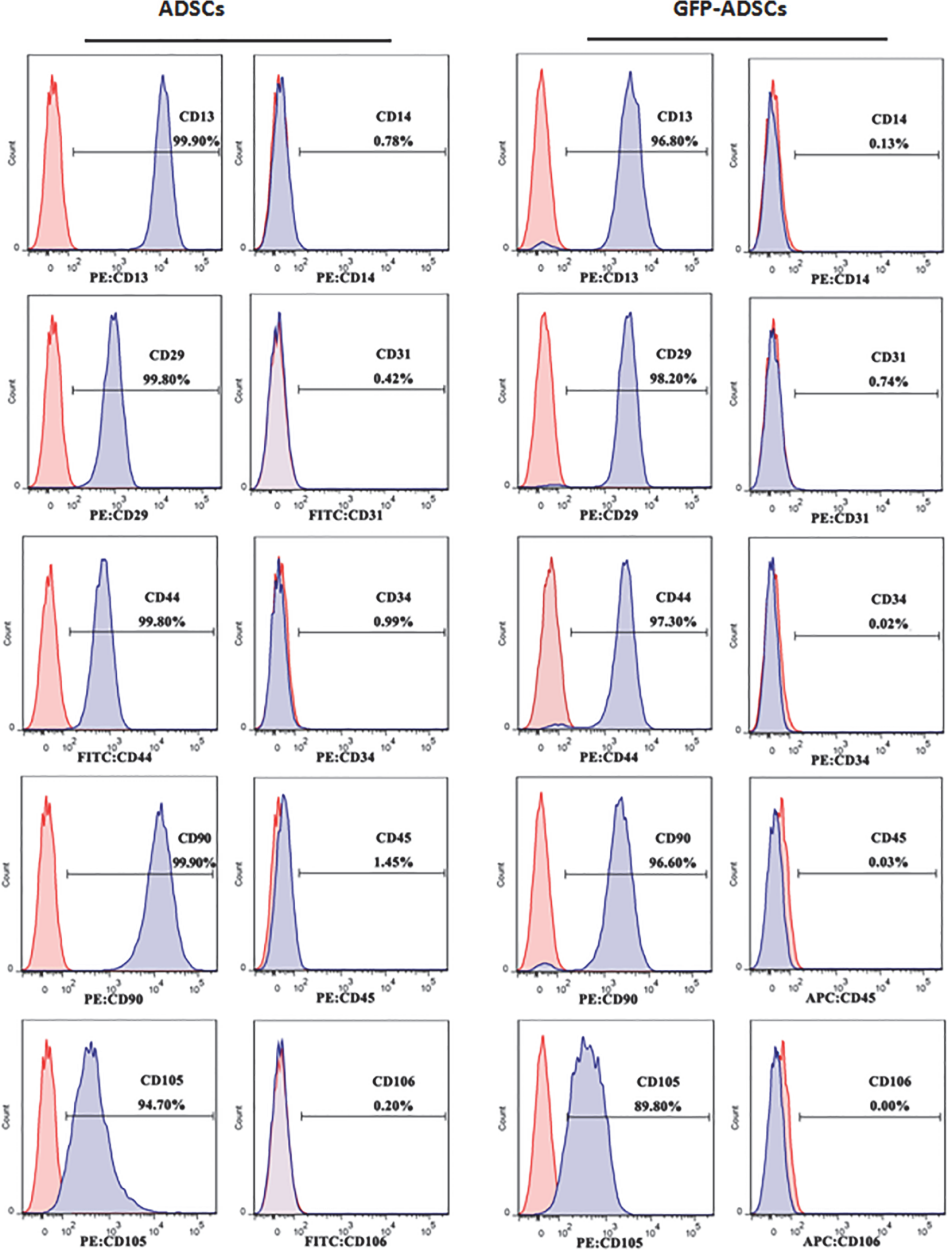
Detection of cell surface antigens. Cell surface antigens of GFP-expressing ADSCs and control ADSCs were detected with flow cytometry. Nonspecific IgG was served as the control of background fluorescence. The positive percentage of cell surface was showed in the histograms.

As shown in Fig 3, high levels of CD marker CD29 (98.20%), CD44 (97.30%), CD90 (96.60%), and CD105 (89.80%) in GFP-expressing ADSCs after transduction were displayed, and they were considered as the markers for mesenchymal stem cells [30]. Similarly, these high levels of CD markers also could be detected in the control ADSCs. In contrast, the positives percentage of hematopoietic lineage markers CD31, CD34, and CD45 were under 2.0% in the GFP-expressing ADSCs or hardly detectable in the control ADSCs. Additionally, the results of flow cytometry analysis also showed a high level of CD13 expression and the absence of CD14 and CD106 expression in the GFP-expressing ADSCs and control cells, consistent with previous reports [31].

### The survival and proliferation of human ADSCs in zebrafish embryos

**Fig 4:**
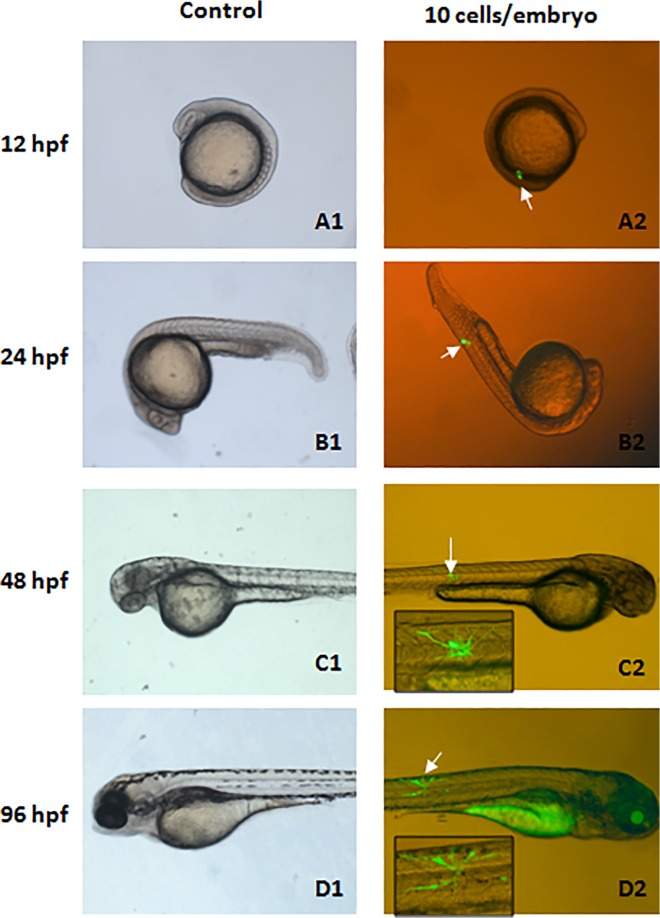
Survivals of human ADSCs transplanted into zebrafish embryos. The GFP-expressing human ADSCs were transplanted into zebrafish embryos at 3.3–4.3 hpf after their chorions were removed, and followed by observation under fluorescence microscope at different time points. Each embryo was administered with about 10 cells, but the control did not receive cell transplantation and only their chorions were removed. (A1-D1) The representative control images of a same embryo standing for typical development stages at 12, 24, 48, and 96 hpf (bright). (A2-D2) The representative images of a same embryo captured under fluorescence microscope at indicated time points (bright + fluorescence). Original magnification: 40 ×. The white arrows indicate the location of GFP-expressing ADSCs (green) in the embryos and zebrafish. The insets in (C2) and (D2) show the enlarged images of transplanted GFP-expressing cells in the zebrafish. hpf: hour post-fertilization.

**Fig 5:**
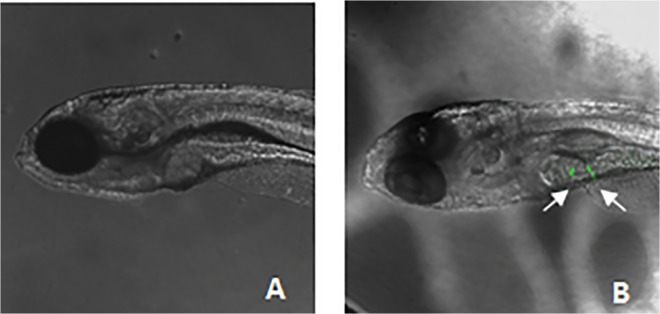
GFP-expressing ADSCs in zebrafish were observed by using laser confocal fluorescence microscope. Human ADSCs expressed GFP were xenotransplanted into zebrafish embryos at 3.3–4.3 hpf after their chorions were removed, and followed by observation under laser confocal fluorescence microscope at indicated time. Each embryo was injected with about 10 cells, and the control did not receive cell transplantation. (A) The control did not demonstrate any green fluorescence. (B) The representative image captured under a laser confocal fluorescence microscope at 15 dpf, displayed the GFP distributions (white arrows indicated) of transplanted cells in the zebrafish. Original magnification: 100 ×.

A total of 200 zebrafish embryos were transplanted with GFP-expressing human ADSCs, and 178 embryos at 24 hpf were alive after transplantation (survival rate 89%). Another 200 embryos did not receive any transplantation as the controls, but their chorions were removed like the transplanted groups, and 188 embryos were found to be alive at 24 hpf (survival rate 94%). There was no significant difference in survival rates between the groups. GFP-expressing human ADSCs in zebrafish embryos were observed at different time after transplantation. As shown in Fig 4, no fluorescence was observed in the control embryos or fish larva (Fig 4A1–4D1); on the other hand, green dots standing for human ADSCs in embryo (Fig 4A2) or larva (Fig 4B2) could be clearly observed with fluorescence microscopy at 12 or 24 phf after transplantation. With embryo development, green dots became irregular and disperse, but green fluorescence *in vivo* was still observable at 48 (Fig 4C2) and 96 phf (Fig 4D2). With time extension, we found that the green fluorescence *in vivo* could be sustained for more than 15 days (Fig 5). These results suggest that the transplanted human ADSCs can survive in zebrafish embryo and larva.

Additionally, transplantation with 10 ADSCs per embryo did not affect normal development of zebrafish embryo, and there was no significant difference of development between experimental groups and the control groups (Table 1). However, we found that transplantation with more than 30 cells per embryo frequently resulted in more abnormal development of embryos (data not shown).

**Table 1:** Effects of ADSCs transplantation on the development of zebrafish embryos.

**Groups**	**Rate of egg coagulation at 24 hpf (%)**	**Rate of spontaneous movement with 20 s at 24 hpf (%)**	**Normal heart rate at 48 hpf (%)**	**Rate of deformity at 96 hpf (%)**	**Rate of death at 96 hpf (%)**
**Control**	2.2 ± 3.9	94.4 ± 9.6	83.1 ± 5.1	10.0 ± 6.7	12.2 ± 8.4
**10 Cells/embryo**	2.2 ± 1.9	93.3 ± 3.3	88.9 ± 1.9	11.1 ± 5.1	12.2 ± 10.1

GFP-expressing ADSCs were transplanted into zebrafish embryos at 3.3–4.3 hpf after their chorions were carefully removed. The control did not receive cell transplantation, but their chorions were also removed. Specific assessment parameters at different time points listed in the table were detected.

Values are presented as means ± SD, and statistical analysis was analyzed by using Student’s *t* test to compare experimental groups with the control groups.

*p* < 0.05 is defined as statistical significance. The results demonstrated that the transplantation with 10 human ADSCs per embryo did not affect the normal development of zebrafish embryos.

**Fig 6:**
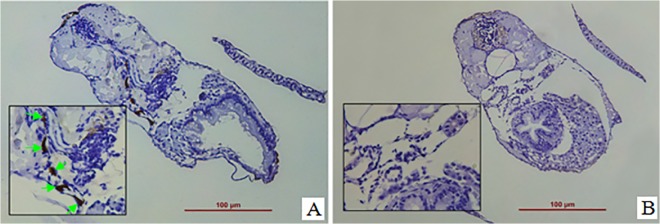
The proliferation of transplanted ADSCs detected by immunohistochemical staining. The GFP-expressing human ADSCs were transplanted into the zebrafish embryos at 3.3–4.3hpf. The proliferation of transplanted cells were evaluated by immunohistochemical staining with rabbit anti-human Ki-67 monoclonal antibody at 6 days post fertilization. (A) Representative image of immunohistochemical staining demonstrated that the proliferating cells were Ki-67 positive staining (brownish, indicated by green arrow), suggesting that the transplanted human ADSCs could proliferate in the zebrafish. (B) The control that did not receive cell transplantation, was negative staining of Ki-67. Scale bar: 100 μm.

**Fig 7:**
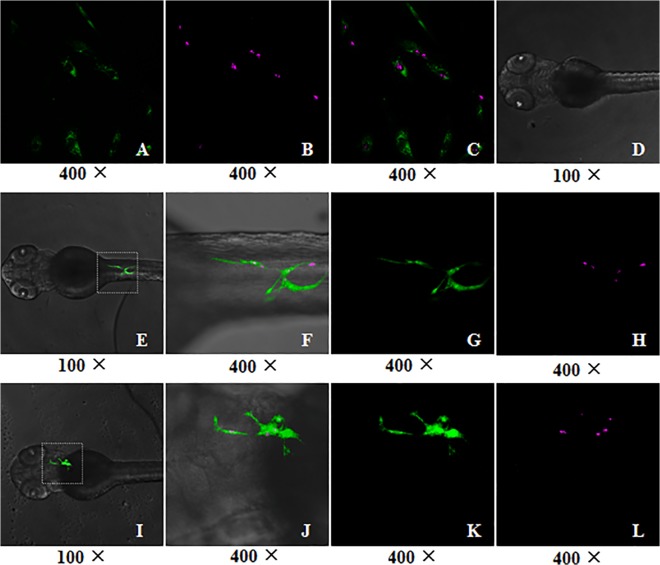
The proliferation of transplanted ADSCs detected by immunofluorescence staining. The GFP-expressing human ADSCs were transplanted into the fish embryos at 3.3–4.3 hpf and the immunofluorescence of whole-mount zebrafish larvae by using Ki-67 staining was performed as described in the *materials and methods*. The microphotos of the ADSCs or zebrafish larvae were captured by laser confocal scanning microscope. (A-C) The representative images of GFP-expressing human ADSCs seeded on the glass slides, and (A) indicates the cells expressed GFP (green), and (B) indicates the positive staining of Ki-67 (red). (C) is the merged image of (A) and (B). (D) The control zebrafish larva, which did not receive any transplantation, does not show green or red fluorescence. (E) and (I) Two representative fish larvae received the transplantation of GFP-expressing human ADSCs, show the different distributions of ADSCs in the fish. (F) and (J) are respectively the higher power images of the outlined areas in (E) and (I). (F) is the merged image of (G) and (H) under bright field; and (J) is the merged image of (K) and (L). (G) and (K) indicate the transplanted ADSCs expressed GFP (green). (H) and (L) demonstrate the positive staining of Ki-67 in the fish (red), indicating the proliferation of transplanted ADSCs in the fish.

Immunohistochemical staining by using rabbit anti-human Ki-67 monoclonal antibody indicated that the proliferating human ADSCs *in vivo* were Ki-67 positive staining (brownish, indicated by green arrow) (Fig 6A) at 6 days post fertilization, but no Ki-67 positive cells were observed in fish cells and the control zebrafish (Fig 6B). Moreover, as shown in Fig 7, immunofluorescence staining of whole-mount zebrafish further confirmed that the Ki-67 positive cells were those transplanted human cells which demonstrated green and red fluorescence in the merged images. These data suggest that the transplanted human ADSCs could proliferate in zebrafish.

### Detection of CD105 and CD31 expression of human ADSCs in zebrafish embryos

**Fig 8:**
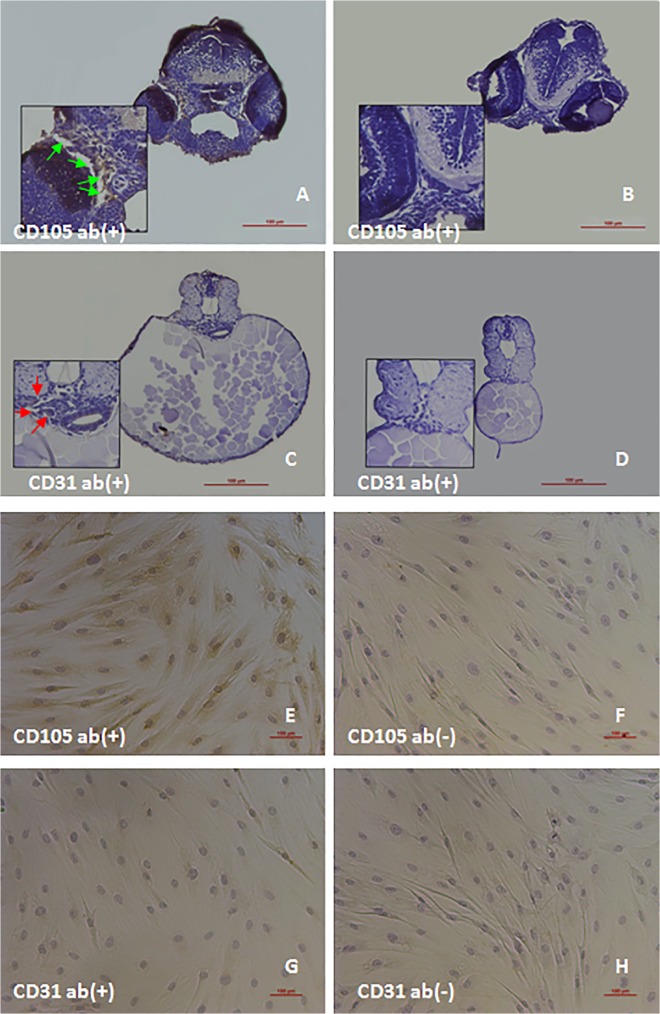
Detection of CD105 and CD31 expression of ADSCs. Human ADSCs were xenotransplanted into the zebrafish embryos at 3.3–4.3hpf, and immunohistochemical staining and immunocytochemical staining were used to evaluate the expression of CD105 and CD31 of human ADSCs in the zebrafish at 48 hpf, and in culture dishes. (A-D) Representative images of immunohistochemical staining: the xenotransplanted ADSCs expressed positive CD105(brownish, indicated by green arrow) in the zebrafish embryos (A), and CD105 expression could not be detected in the control without ADSCs transplantation (B); the xenotransplanted ADSCs (indicated by red arrow) did not express CD31 antigen in the zebrafish embryos (C), and CD31could not be detected in the control without ADSCs transplantation (D). (E-H) Representative images of immunocytochemical staining: CD105 expression was detectable in the ADSCs cultured in the dishes before transplantation (E), and negative control for CD105 (without the addition of primary antibody) (F); CD31 expression was not detectable in the ADSCs cultured in the dishes before transplantation (G) and in the negative control (H). Scale bar: 100 μm. ab(+): added primary antibody; ab(-): no primary antibody.

**Fig 9:**
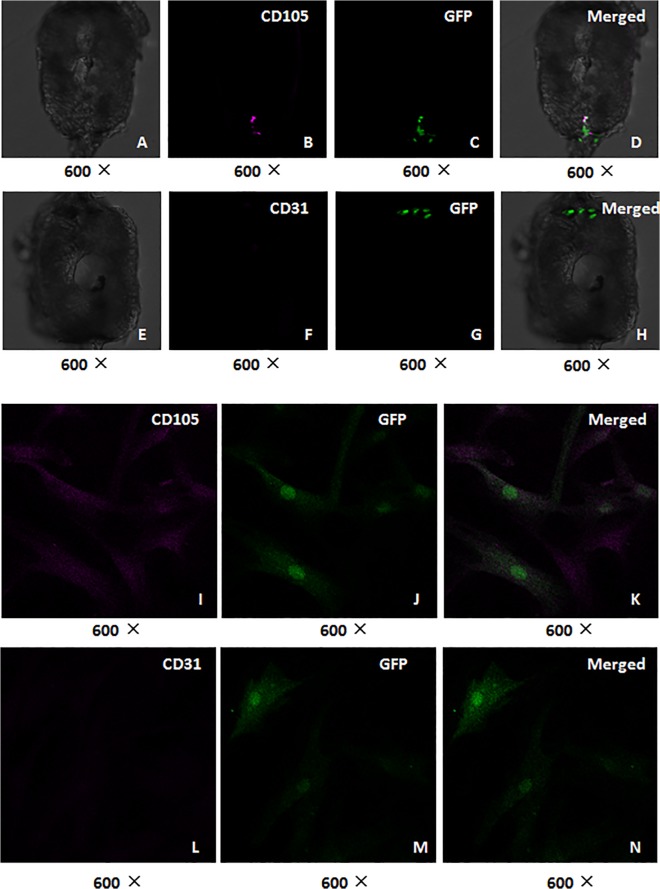
The transplanted ADSCs express CD105, but not CD31 in zebrafish. The GFP-expressing human ADSCs were xenotransplanted into the zebrafish embryos at 3.3–4.3hpf, and immunofluorescence staining was performed to detect CD105 and CD31 expression of human ADSCs in the zebrafish at 48 hpf, and the images were captured with laser confocal scanning microscope. A-H: Representative images of immunofluorescence staining for zebrafish tissue sections. (A-D) Co-localization of CD105 and GFP in the transplanted ADSCs: (A) Bright field; (B) CD105 was positive; (C) GFP was positive; (D) Merged (A), (B) and (C). (E-H) The co-localization of CD31 and GFP in the transplanted ADSCs: (E) Bright field; (F) CD31 was negative; (G) GFP was positive; (H) Merged (E), (F) and (G). (I-N) Representative images of immunofluorescence staining for the ADSCs seeded on the glass slides. (I-K) The co-localization of CD105 and GFP in the human ADSCs before their transplantation: (I) CD105 expression was positive; (J) GFP was positive; (K) Merged (I) and (J). (L-N) The co-localization of CD31 and GFP in the human ADSCs before their transplantation: (L) CD31expression was negative; (M) GFP was positive; (N) Merged (L) and (M).

Immunohistochemical staining analysis indicated that cell surface antigen CD105 expression was observed in the xenotransplanted human ADSCs in zebrafish embryos at 48 hpf, and the human ADSCs cultured in the dishes also expressed CD105. However, CD31expression could not be detected in the recipient zebrafish and cultured ADSCs in the dishes (Fig 8). The immunofluorescence staining for the detection of CD105 and CD31 expression further confirmed that the transplanted ADSCs in the zebrafish could express CD105, but not CD31 (Fig 9). These results seem to suggest that human ADSCs can keep their property and maintain the state of undifferentiation in a short term.

## Discussion

We successfully obtained human ADSCs from donors by a rapid and efficient method as previously described [25]. These cells demonstrated spindle and fibroblast-like shape (Fig 2A–2D), and proliferated rapidly *in vitro* under appropriate culture conditions. To track the fate of human ADSCs transplanted into zebrafish embryos, we used a lentiviral vector system containing pLVX-mCMV-ZsGreen-Puro vector to transduce GFP cDNA gene into these cells, and obtained GFP-expressing ADSCs through screening with addition 1 μg/mL of puromycin in culture medium. These GFP-expressing cells maintained strong cell viability and expanded rapidly, and there was no significant difference in cell growth between the transduced cells and control group (Fig 2E). Furthermore, exogenous GFP expression in ADSCs hardly affected the expression levels of cell surface antigens (Fig 3), which was consistent with previous studies [29–30, 32].

Investigation of stem cell biology in recipient animal is essential before attempting clinical therapy of stem cells. Therefore, this requires development of a suitable animal model for *in vivo* research before clinical application. In this study, we have established a zebrafish model for cell xenotransplantation, and this model has the advantages such as rapid development and transparency of embryos, short breeding cycle, and established abundant strains for various genetic disorders. The early-stage zebrafish embryos are immunologically immature, providing good tolerance of cell xenotransplantation and an acceptable model to investigate the biology of stem cells following their transplantation. Additionally, we recognize that our research has some limitations. Firstly, we used zebrafish embryos at 3.3–4.3 hpf to be recipient of xenotransplanted cells, and they were very tiny and had no enough space to accommodate numerous xenotransplanted cells. Our preliminary trial indicated that cell doses of more than 30 cells/embryo could result in a significant increase in the abnormal development or mortality of zebrafish embryos. In this study, we found that cell doses of 10 cells/embryo was appropriate and did not affect the survival of injected embryos. With regard to the limited quantity of transplanted cells in the embryos, it was hard to find which slices that contained the transplanted cells when immunohistochemical staining was performed. Additionally, in the present study, we did not research the role of transplanted ADSCs during the development of recipient zebrafish embryos. Finally, although we did evaluate the differentiation of xenotransplanted ADSCs, we only detected the expression of CD105 and CD31 of human ADSCs in the recipient zebrafish embryos at 48 hpf. There, more CD markers and long term differentiation of human ADSCs after xenotransplantation need to be elucidated in future work.

In the present study, human ADSCs were transplanted into zebrafish embryos by direct injection under a microstereoscope. Many important technical details for cell transplantation in this experimental system should be noted for the success of this procedure, because the size of zebrafish embryos is very small and the glass needles used to transplant cells are intrinsically fragile, operators need to be careful and skillful to inject cells into embryos, and need to minimize the damage to the embryos. Additionally, removal of embryo chorion contributes to improving the efficiency of cell injection, but does not affect normal development of embryos (data not shown). Regular change of culture medium and maintenance of the temperature at 28°C after transplantation are important to prevent abnormal development or embryonic death. According to our observation, the embryos with strong viability and high quality are the most important factor for a successful engraftment of human ADSCS. In the present study, observable green fluorescence of GFP expressed in transplanted human ADSCs and positive staining of human Ki-67 have demonstrated that human ADSCs transplanted into zebrafish embryos have potential to survive and proliferate with the development of zebrafish embryos and larvae (Figs 4–7). We found that once successful transplantation of human ADSCs and subsequent normal development of embryos were established, the donor cells in host tissues could be maintained for a long term (more than three months, data not shown). However, a dramatic reduction or even complete loss of donor cells with time extension was reported in several xenogeneic transplantation studies in animal models of rat, mouse, goat, and pig [9, 33–35]. We speculate that the mature fish developed from the embryos injected with donor cells mistake these xenogeneic cells for their own components, and therefore maintained them for longer time than other animals. Additionally, immune-response system of zebrafish embryos at early stage including 3.3–4.3 hpf is seriously immature, which undoubted contributes to the survival and proliferation of donor cells. On the other hand, we detected the expression of CD105 and CD31of human ADSCs after xenotransplantation. The results indicated that these xenotransplanted ADSCs could still express CD105 and did not express CD31, similar their expression before transplantation, which seemed to imply that human ADSCs could maintain their status of undifferentiation in a short term. As for our knowledge, in our current study, we were able to perform stem cell xenotransplantation in zebrafish embryos at the earliest-stage (3.3 hpf). Thus, the donor stem cells are easier to engraft in the host. Previous studies have indicated that heterogeneity of the transplanted cell population might result in its loss in recipients if various cell types have different development potentials or replication capacity [36]. In addition, we found that the presence of green fluorescence of GFP expressed in human ADSCs mainly located in the position in which cells were initially delivered. It is pointed out that whether these transplanted ADSCs undergo cellular fusion or adaptation upon engraftment into recipient tissues remains unclear, and needs to be studied in further work.

In conclusion, we successfully established a zebrafish model for efficient xenogeneic cell transplantation. The xenotransplanted human ADSCs not only can survive and proliferate in the zebrafish embryos and larvae after cell xenotransplantation in zebrafish embryos at the early stage, but also seem to maintain their undifferentiation status in a short term. Therefore, this xenograft model of zebrafish is possibly a promising technical platform for the study of human stem cell biology and physiology *in vivo*.
